# Editorial: Roles of Chondroitin Sulfate and Dermatan Sulfate as Regulators for Cell and Tissue Development

**DOI:** 10.3389/fcell.2022.941178

**Published:** 2022-06-08

**Authors:** Shuji Mizumoto, Jessica C. F. Kwok, John M. Whitelock, Fuchuan Li, Roberto Perris

**Affiliations:** ^1^ Department of Pathobiochemistry, Faculty of Pharmacy, Graduate School of Pharmacy, Meijo University, Nagoya, Japan; ^2^ Faculty of Biological Sciences, School of Biomedical Sciences, University of Leeds, Leeds, United Kingdom; ^3^ Centre for Reconstructive Neuroscience, Institute of Experimental Medicine, Czech Academy of Sciences, Prague, Czech Republic; ^4^ Graduate School of Biomedical Engineering, University of New South Wales, Adelaide, NSW, Australia; ^5^ National Glycoengineering Research Center, Shandong Key Laboratory of Carbohydrate Chemistry and Glycobiology, NMPA Key Laboratory for Quality Research and Evaluation of Carbohydrate-Based Medicine, Shandong University, Jinan, China; ^6^ Department of Chemical and Life Sciences and Environmental Sustainability, University of Parma, Parma, Italy

**Keywords:** chondroitin sulfate, dermatan sulfate, proteoglycan, cell signal, stem cell, tissue morphogenesis

Chondroitin sulfate (CS) and dermatan sulfate (DS) are highly conserved glycosaminoglycans (GAGs) known to mediate many of the functions that proteoglycans (PGs) exert in a myriad of biological events taking place during embryogenesis, in the adult organism and in pathological conditions. CS was originally isolated from cartilage as early as at the end of the 19^th^ century (Fischer and Boedeker, 1861), albeit its structural properties were resolved decades later (Levene and La Forge, 1913) and its complex sulfation patterns and domain arrangement remain a great challenges in this field. DS was first isolated from porcine skin by Karl Meyer in 1940s (Meyer and Chaffee, 1941) and was much later on found to share some of the characteristics of CS. As all sulfated GAGs, CS/DS chains are covalently attached to serine residue(s) of the core proteins of PGs through a specific and highly conserved GAG-protein tetrasaccharide linkage region encompassing glucuronic acid–galactose–galactose–xylose-(serine) (Lindahl and Rodén, 1966). In 1950s, the first CS-bearing PGs were isolated from bovine nasal septum (more specifically, aggrecan; Mathews and Lozaityte, 1958), while their counterpart first degrading enzyme, “chondroitinase ABC”, was isolated from Proteus vulgaris (Dodgson and Lloyd, 1958).

The cloning of mammalian chondroitin synthases, DS-epimerases and chondroitin sulfotransferases significantly contributed to the gaining of a better understanding of the biosynthesis and homeostatic roles of CS/DS, with particular reference to the action of cytokinesis, the control of bone development, the assembly of collagen fibrils, and the regulation of cytokines as well as chemokines. These discoveries were followed by correlations between CS/DS synthesis and human genetic disorders, including spondyloepiphyseal dysplasia with congenital joint dislocations, Temtamy preaxial brachydactyly syndrome, and Ehlers-Danlos syndrome.

By combining the knowledge on the biosynthetic and catalytic activity of CS/DS enzymes and the use of transgenic technologies it has been possible to correlate the phenotypic traits of the brachymorphic mouse to a disturbed sulfation of CS of cartilage matrix PGs, as well as reconcile these synthetic GAG deficits to genetic abnormalities in humans. This transgenic mouse provided the first model for addressing the *in vivo* function of CS in that specific tissue and paved the way for generating numerous other mouse mutants with altered expression of key CS/DS regulating molecules. It was, however, through the production of the first immunological tools that analyses of the distributional, structural and functional traits of CS/DS and their isoforms became more readily accomplishable. The pioneering, milestone work of Bruce Caterson and John Couchman (Couchman et al., 1984) opened this field and their work was soon followed by the impactful, but unfairly overlooked, studies of Michael Sorrell who was able to generate an array of monoclonal antibodies recognizing subdomains of the intact CS and DS chains (Sorrell et al., 1990).

Through the advent of more powerful technologies, including oligosaccharide libraries, microarrays, and sophisticated proteomic approaches, it has been possible delineate the complexity of the domain organization of CS, DS, and CS/DS hybrid chains and consequently gain a better understanding of the dynamics and structural-functional details of the molecular interactions that these GAGs, “wobble motifs”, engage with a spectrum of proteins (Purushothaman et al., 2012).

Collectively, decades of studies on the structural properties and cellular and molecular functions of CS and DS have highlighted their importance in an ample spectrum of biological phenomena and have incited further investigations to be pursued to fully unveil their biological significance. This special issue was therefore proposed to expand our vision on what is currently known about the role of CSs and DSs in biological processes, spanning from those regulating embryonic development to those maintaining a proper tissue homeostasis, and on the methods and technologies currently used to analyse their structural-functional properties.

A study reported in this issue by Schaberg et al., highlights that in addition to the well-established function of heparan sulfate (HS) in growth factor signaling, CS may similarly participate in the modulation of fibroblast growth factor 2 (FGF2)-dependent cell-cycle progression in spinal cord neural stem and progenitor cells. Alongside this contribution, the article by Ogura and Nishihara describes how dermatan 4-*O*-sulfotransferase-1 (D4ST1) may control the differentiation state of murine embryonic stem cells, emphasizing how DS-PGs and D4ST1, as a promoter of 4-*O*-sulfation in DS-like HS, may influence signaling pathway(s) associated with cell differentiation. Comprehensively, these studies emphasize a critical role of CS, DS, and the PGs bearing such GAGs in the control of stem/progenitor cell development.

A parallel study by Nitahara-Kasahara et al., further demonstrates that D4st1 knockout mice display a myopathy-related phenotype including variation in fiber size and altered distribution in the muscle interstitium, resulting in a decreased exercise capacity. This finding suggests that D4ST1, and/or the DS-PGs affected by its deletion, regulate skeletal muscle myogenesis and muscle physiology, and highlights potential therapeutic approaches to be undertaken in patients of musculo-contractural Ehlers-Danlos syndrome caused by mutations in D4ST1. In their review, Mizumoto and Yamada elegantly summarize what we have learned through the use of CS/DS-deficient mice and how such mutants may be further exploited to improve our understanding of the role of the GAGs during both embryonic development and adult life.

Schwartz and Domowicz have reviewed in this issue the roles of CS-bearing PGs as regulators of skeletal development and as primary PGs affected by GAG-related disorders such as mucopolysaccharidoses and osteoarthritis. These authors specifically discuss the Indian hedgehog, BMPs, FGFs, and TGFs signaling pathways responsible for growth plate development, which are underlined to be elicited through the interaction of the growth factors with the CS chains of aggrecan, the prototype CS-bearing PG that may carry up to 100 such chains. Their essay provides a comprehensive view on the importance of aggrecan and other early expressed CS-bearing PGs during tissue development and the morphogenetic events leading to the formation of the specialized connective tissue structures building our skeleton.

Nadanaka et al., have contributed with an appealing article demonstrating that knockout of chondroitin 4-*O*-sulfotransferase-1 (C4ST1) in breast cancer cell lines results in the lack of CS side chains on syndecan-1, which thereby inhibits the cell surface shedding of sundecan-1 and causes a reduced rate of cell proliferation via the action of a small ubiquitin-like modifier of Akt. The observations would therefore imply that therapeutic targeting of C4ST1, as well as inhibited cleavage of syndecan-1, may be exploited for halting progression of breast cancer. In addition, the study proposed by Maciej-Hulme provides evidence for a specific effect hyaluronidase-4 (HYAL4), which specifically degrades CS but not hyaluronan, in cancer progression. Thus, the findings documented in these two articles strongly support the role attributed to CS in cancer development and suggest that alterations in CS distribution may be a key element to consider when addressing the biology of cancer and the search for novel diagnostic and therapeutic targets.

A perineuronal net is a layer of a lattice-like matrix enwrapping the surface of the soma and dendrites of neurons of the CNS. The perineuronal nets are mainly composed of CS-bearing PGs, which uniquely comprehend an array of aggrecan isoforms, hyaluronan, link proteins (HAPLNs), and tenascin-R. The contribution by Nojima et al., provides evidence that, at the calyx of Held synapses, HAPLN4 favors the perineuronal localization of brevican, but not that of aggrecan. Hence, the findings suggest that distinct HALPNs may regulate the localization of specific CS-bearing PGs, such as to finetune the formation and ultimate composition of neuronal class-specific perineuronal nets.

The work of Sakamoto et al. provides challenging inputs into the molecular mechanism by which CS influences the formation of dystrophic endbulbs, which are swollen axonal tips with multiple vacuoles. Upon injury in the CNS, CS-bearing PGs upregulated by the reactive astrocytes bind to receptor protein tyrosine phosphatase sigma (RTPTσ) to dephosphorylate cortactin, which then stabilizes actin microfilaments to facilitate lysosome autophagy. This finding may provide a novel view on therapeutic strategies to adopt for inducing axonal regeneration. As a corollary, Hayes and Melrose comprehensively review the function of a variety of neuronal CS/DS-PGs in the formation and morphogenesis of the CNS, by also discussing the cell-surface ligands and putative receptors for CS-PGs, such as RTPTσ, contactin-1, semaphorins, and Nogo.

The study provided by Noborn et al., describes the recent advances in defining the CS glycoproteome using reversed phase nano-liquid chromatography-tandem mass spectrometry (nLC-MS/MS) to enabled the discovery of novel CS-PGs. This contribution provides further evidence of the complexity of CS/DSs and further emphasizes the powerfulness of mass spectrometric approaches to harness the dissection of this complexity.

Quantitative determination of the expression levels glycosyltransferases, sulfotransferases, and epimerases responsible for the biosynthesis of GAGs bound to PGs may provide valuable information on the resulting overall abundance of GAGs that may be bound to their partner PGs, as well as on the degree and variability of the sulfation patterns of these moieties. Huang et al., report the application potential of a recently developed, comprehensive glycosylation mapping tool based on gene expression profiles from RNA-seq data, GlycoMaple, designed to enable the visualization and assessment of glycan structures (Huang et al., 2021). The authors also demonstrate that the expression levels of genes encoding constituents of the CS/DS biosynthetic pathways that were established though the tool for normal brain, pancreatic tumors and breast cancer were consistent with those previously published through biochemical means. These findings suggest that new insight into GAG profiles may be gathered for various human diseases through the use of the GlycoMaple software tool, provided that the supportive RNAseq data is available.

Finally, Zhang and Chi provided a very informative review the known CS/DS-protein interaction that may take place in different disease conditions, including tumor growth and metastasis formation, nerve tissue repair, virus infection, and atherosclerosis. They also describe the analytical tools that may be employed for the characterization of these interactions.

Conclusively, we hope that the collection of articles embodied in this special issue will be useful for researchers of the field and scientists of other fields who may appreciate acquiring information about the biological and pathological importance of CS and DS, as well as the PGs carrying such GAGs. We are particularly thankful to all authors of these interesting and stimulating articles and would also like to acknowledge the splendid work of the Editorial and Editorial Staff in compiling the Topic Issue.
